# Iridoids from *Canthium subcordatum iso*-butanol fraction with potent biological activities

**DOI:** 10.1186/s12906-016-1536-8

**Published:** 2017-01-05

**Authors:** Christelle Joubouhi, Jean-de-Dieu Tamokou, David Ngnokam, Laurence Voutquenne-Nazabadioko, Jules-Roger Kuiate

**Affiliations:** 1 Department of Chemistry, Laboratory of Environmental and Applied Chemistry, Faculty of Science, University of Dschang, P.O. Box 67 Dschang, Cameroon; 2 Department of Biochemistry, Laboratory of Microbiology and Antimicrobial Substances, Faculty of Science, University of Dschang, P.O. Box 67 Dschang, Cameroon; 3 Groupe Isolement et Structure, Institut de Chimie Moléculaire de Reims (ICMR), CNRS UMR 7312, Bat. 18 BP.1039, 51687, Reims cedex 2, France

**Keywords:** *Canthium subcordatum*, Rubiaceae, Fruit extracts, Phytochemical analysis, Iridoids, Antimicrobial, Antioxidant

## Abstract

**Background:**

The continuous emergence of multi-drug-resistant bacteria drastically reduces the efficacy of antibiotic armory and, consequently, increases the frequency of therapeutic failure. The discovery of new antibacterial drugs is an urgent need. The present study reports the antibacterial and antioxidant activities of the methanol extract, fractions and iridoids from *Canthium subcordatum,* a plant traditionally used as antidiabetic, anti-inflammatory, and antimicrobial.

**Methods:**

Broth microdilution assay was used to determine minimum inhibitory concentrations (MICs) and minimum bactericidal concentrations (MBCs) of extracts and iridoids against *Staphylococcus aureus, Vibrio cholerae* and *Shigella flexneri.* Antioxidant activity was evaluated using 1,1-diphenyl-2-picrylhydrazyl (DPPH) and gallic acid equivalent antioxidant capacity (GAEAC) assays. The samples were also tested for their cytotoxicity against human red blood cells (RBC).

**Results:**

The methanol extract, hexane, ethyl acetate and *iso-*butanol fractions from *C. subcordatum* fruits displayed different degrees of antioxidant (EC_50_ = 62.83–70.17 μg/ml; GAEAC = 45.63–58.23 μg/ml) and antibacterial (MIC = 128–512 μg/ml) activities. Canthiumoside 1(**1**) and linearin (**7**) were the most active antioxidant (EC_50_ = 1.12–2.03 μg/ml; GAEAC = 79.82–92.35 μg/ml) and antibacterial (MIC = 8–64 μg/ml) compounds while the most sensitive bacterium was *Staphylococcus aureus*. The tested samples were non-toxic to normal cells.

**Conclusion:**

Our results demonstrated that compounds **1** and **7** were potent antibacterial agents and DPPH/ABTS·^+^ radical scavengers, so they warrant further investigation.

## Background

The uses of plants in the indigenous cultures of developing countries are numerous and diverse. For many people, the high cost of imported conventional drugs and/or inaccessibility to western health care facilities has led to overreliance on traditional medicine since it is affordable and available to rural people. On the other hand, even when western health facilities are available, traditional medicine is viewed as an efficient and an acceptable system from a cultural perspective [1, 2]. Oxidative stress and diarrheal diseases are among some of the indications treated using traditional remedies in Cameroon. Diarrheal disease is a leading cause of child mortality and morbidity in the world due to various factors such as the HIV/AIDS pandemic, poor hygiene, overcrowding and resistance to conventional antibacterials while oxidative stress can lead to many illnesses including cardiovascular diseases, diabetes, inflammation, degenerative diseases, cancer, anemia, and ischemia [3]. Among the diarrheal diseases, cholera is a serious epidemic disease caused by the gram-negative bacterium *Vibrio cholerae* [4]. *Vibrio cholerae*, serotypes O1 and O139 has ability to produce an enterotoxin, cholera toxin that is a major determinant of virulence for cholera. There is a consensus among the scientific community that plant derived products have been playing a dominant role in the discovery of leads for the development of drugs for the treatment of human diseases [3]. *Canthium subcordatum* (formely *Psydrax subcordata*) belonging to Rubiaceae family is a tree which grows in central and western Africa and reaches a height of more than 10 m [5]. Its roots, leaves and stem bark are used for medicinal purposes [6]. Alcoholic extracts of the stem bark have potential antidiabetic properties [6] and roots were used to treat malaria fever, inflammation and cardiovascular disease [7]. Petroleum ether and dichloromethane extracts of *C. subcordatum* have shown anti-inflammatory activity in COX-1 and COX-2 assays [8]. Previous phytochemical works on this plant species and other *Canthium species* revealed the presence of iridoids [8–10], iridoid peptidic alkaloids [11, 12], flavonoids [10], terpenoids and miscellaneous [12, 13]. However, it is not yet known which of the phytoconstituents is responsible of the antimicrobial effect of this plant, when it is used to cure infectious diseases and oxidative stress conditions. Therefore, the present study reports the antibacterial and antioxidant activities of extracts and iridoids from *C. subcordatum* fruits.

## Methods

### Plant material

The fruits of *Canthium subcordatum* DC (syn. *Psydrax subcordata* DC Bridson) were collected in Foto village (Menoua Division, Western region of Cameroon), in April 2012. Authentication was performed by Victor Nana, a Botanist of the Cameroon National Herbarium, Yaoundé, where a voucher specimen (N° 19579/SRF/CAM) has been deposited.

### Experimental

The melting point, optical rotation, IR, ^1^H NMR, ^13^C NMR, COSY, NOESY, HSQC, HMBC and HR-TOFESIMS experiments were performed as previously described [10].

### Extraction and isolation

**Fig. 1:**
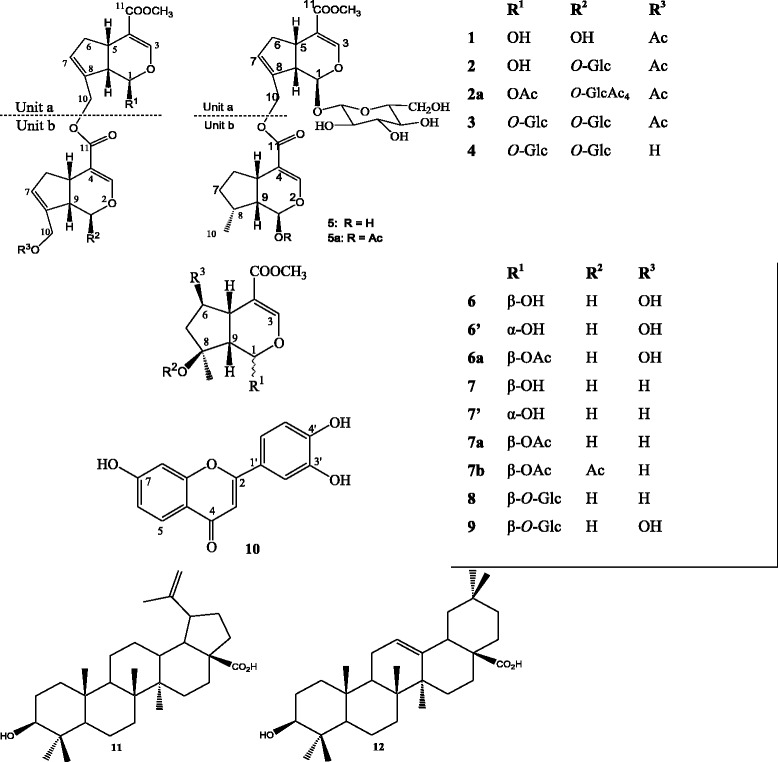
Chemical structures of the isolated compounds (**1**–**12**). **1:** canthiumoside 1; **2:** canthiumoside 2; **3:** canthiumoside 3; **4:** canthiumoside 4; **5:** canthiumoside 5; **5a:** canthiumoside 5a; **6:** shanzhigenin methyl ester; **6’:** 1-epishanzhigenin methyl ester**; 7:** linearin; **7’:** 1-epilinearin; **8:** mussaenoside; **9:** shanzhiside methyl ester; **10:** 3’,4’,7- trihydroxyflavone; **11:** betulinic acid; **12:** oleanolic acid

The *iso*-butanol, ethyl acetate and hexane fractions as well as the isolated compounds were obtained as previously described [10]. Briefly, the dried fruits of *C. subcordatum* (3.50 kg) were extracted with MeOH, and the resulting crude extract was suspended in water and successively extracted with *n*-hexane, ethyl acetate and *iso-*butanol to yield hexane, ethyl acetate and *iso*-butanol fractions. The hexane and *iso-*butanol-solute fractions were further fractionated and purified over silica gel column chromatography to yield compounds **11**–**12** and **1**–**10** respectively [10]. None compound has been isolated after the fractionation of the ethyl acetate fraction. Structure elucidation by spectroscopy techniques showed that they are canthiumosides 1–4 and 5a (**1**–**4**, **5a**), shanzhigenin methyl ester (**6**) and 1-epishanzhigenin methyl ester (**6**’), linearin (**7**) and 1-epilinearin (**7**’), mussaenoside (**8**), shanzhiside methyl ester (**9**), 3’,4’,7-trihydroxyflavone (**10**), betulinic acid (**11**), and oleanolic acid (**12**) (Fig. 1).

### Antibacterial assay

#### Microorganisms

A total of six bacterial strains were tested for their susceptibility to compounds and these strains were taken from our laboratory collection (kindly provided by Dr. T. Ramamurthy, NICED, Kolkata). Among the clinical strains of *Vibrio cholerae* used in this study, strains NB2 and SG24(1) belonged to O1 and O139 serotypes, respectively. These strains were able to produce cholera toxin and hemolysin. The other strains used in this study were *V. cholerae* non-O1, non-O139 (strains CO6 and PC2); and *Shigella flexneri* SDINT. The *V. cholerae* non-O1 and non-O139 strains, were positive for hemolysin production but negative for cholera toxin production. The strains of *V. cholerae* and *S. flexneri* included in the present study were MDR clinical isolates and these were resistant to commonly used drugs such as ampicillin, streptomycin, tetracycline, nalidixic acid, furazolidone, co-trimoxazole, etc. A reference strain, *Staphylococcus aureus* ATCC 25923, was used for quality control. The bacterial strains were maintained on agar slant at 4 °C and subcultured on a fresh appropriate agar plates 24 h prior to any antibacterial test. The Mueller Hinton Agar (MHA) was used for the activation of bacteria. The Mueller Hinton Broth (MHB) and nutrient agar (Hi-Media) were used for the MIC and MBC determinations respectively.

### Determination of minimum inhibitory concentration (MIC) and minimum bactericidal concentration (MBC) 

MIC values were determined by a broth micro-dilution method as described earlier [14] with slight modifications. Each test sample was dissolved in dimethylsulfoxide (DMSO) and the solution was then added to Mueller Hinton Broth (MHB) to give a final concentration of 1024 μg/ml. This was serially diluted twofold to obtain a concentration range of 0.50–1024 μg/ml. Then, 100 μl of each concentration was added in each well (96-well microplate) containing 95 μl of MHB and 5 μl of inoculums (at 1.5 ×  10^6^ CFU/ml) for final concentrations varying from 0.25–512 μg/ml. Dilutions of Ciprofloxacin and Ampicillin (256 – 0.125 μg/ml) served as positive controls, while broth with 5 μL of DMSO was used as negative control. The plates were covered with sterile lids, then the contents of each well were mixed using a shaker and incubated at 35 °C for 24 h. The MIC values of samples were determined by adding 50 μl of a 0.20 mg/ml *p*-iodonitrotetrazolium violet solution followed by incubation at 35 °C for 30 min. MIC values were defined as the lowest sample concentrations that prevented this change in color indicating an inhibition of visible growth. Viable microorganisms reduced the yellow dye to a pink color. For the determination of minimum bactericidal concentration (MBC) values, a portion of liquid (5 μl) from each well that showed no growth of microorganism was plated on Mueller Hinton Agar and incubated at 35 °C for 24 h. The lowest concentrations that yielded no growth after this subculturing were taken as the MBC values [15]. All the analyses were carried out in triplicate.

### Antioxidant assay

#### DPPH free radical scavenging assay

The free radical scavenging activity of extracts as well as most of their isolated compounds was performed according to Brand-Williams et al. [16] with slight modifications. Briefly, different concentrations (10 to 2000 μg/ml) of extracts/compounds and vitamin C (positive control) were thoroughly mixed with 3 ml of methanolic DPPH solution (20 mg/l) in test-tubes and the resulting solution was kept standing for 30 min at room temperature before the optical density (OD) was measured at 517 nm. The percentage radical scavenging activity was calculated from the following formula: % scavenging [DPPH] = [(A_0_–A_1_)/A_0_] × 100 [16]. Where A_0_ was the absorbance of the negative control (methanolic DPPH solution) and A_1_ was the absorbance in the presence of the samples. IC_50_ value was determined from the graph obtained using standard vitamin C by using the “y = mx + c” formula from the slope of the graph. All the analyses were carried out in triplicate.

#### Gallic acid equivalent antioxidant capacity (GAEAC) assay

The GAEAC test was done as previously described [17] with slight modifications. In a quartz cuvette, to 950 μl acetate buffer (pH =5.0, 100 mM), the following were added: 20 μl laccase (1 mM stock solution), 20 μl test sample, 10 μl ABTS (2,2’-azinobis(3-ethylbenzothiazoline-6-sulfonic acid) (74 mM stock solution). The laccase were purified from *Sclerotinia sclerotiorum* according to the protocol described [18]. The sample concentrations in the assay mixture were 800, 400, 200, 100, 10 μg/ml for the extracts and 200, 100, 50, 25, 125.5 μg/ml for the isolated compounds. The content of the generated ABTS^●+^ radical was measured at 420 nm after 240 s reaction time and was converted to gallic acid equivalents by the use of a calibration curve (Pearson’s correlation coefficient: *r* = 0.996) constructed with 0, 4, 10, 14, 28, 56, 84 μM gallic acid standards rather than Trolox. Experiments were done in triplicate.

#### Hemolytic assay

Hemolysis test was performed to determine cellular toxicity of the compounds as previously described [19]. Whole blood (10 ml) from a healthy man was collected into a conical tube containing heparin as an anticoagulant (blood group O was used). Extracts (at concentrations ranging from 32 to 2048 μg/ml) and pure compounds (16 to 256 μg/ml), were incubated with an equal volume of 1% human red blood cells in phosphate buffered saline (10 mM PBS, pH 7.4) at 37 °C for 1 h. 1% human red blood cells in buffer was used as a non-hemolytic control whereas buffer containing 1% Triton X-100 and 1% human red blood cells served as a 100% hemolytic control. Cell lysis was monitored by measuring the release of hemoglobin at 540 nm. The assay was repeated thrice. Percent hemolysis was calculated as follows:

$$ \frac{\left[\left(\mathrm{A}595\ \mathrm{of}\ \mathrm{sample}\ \mathrm{treated}\ \mathrm{with}\ \mathrm{compound}\ \hbox{--} \mathrm{A}595\ \mathrm{of}\ \mathrm{sample}\ \mathrm{treated}\ \mathrm{with}\ \mathrm{buffer}\right)\right] \times 100}{\left[\left(\mathrm{A}595\ \mathrm{of}\ \mathrm{sample}\ \mathrm{treated}\ \mathrm{with}\ \mathrm{Triton}\ \mathrm{X}-100\ \hbox{--} \mathrm{A}595\ \mathrm{of}\ \mathrm{sample}\ \mathrm{treated}\ \mathrm{with}\ \mathrm{buffer}\right)\right]} $$

### Statistical analysis

Data were analyzed by one-way analysis of variance followed by Waller-Duncan Post Hoc test. The experimental results were expressed as the mean ± Standard Deviation (SD). Differences between groups were considered significant when *p* <0.05. All analyses were performed using the Statistical Package for Social Sciences (SPSS, version 12.0) software.

## Results and discussion

### Antibacterial activity

In the present work, the extracts as well as 12 compounds isolated from the fruits of *C. subcordatum* were tested for their antibacterial activities against *Vibrio cholerae*, *Shigella flexneri* and *Staphylococcus aureus* (Table 1). The MIC results indicated that the MeOH extract, *n*-BuOH and EtOAc fractions as well as compounds **1**, **2**, **5a** and **6**–**9** inhibited the growth of all tested bacterial species. Hexane fraction and compound **3** showed selective activities, their inhibitory effects being noted on 5 of the 6 (83.33%) tested organisms. The lowest MIC values for the extracts (128 μg/ml) and compounds (8 μg/ml) were recorded on *S. aureus.* The most active extract was the EtOAc extract (MIC = 128–512 μg/ml) while compounds **1** (MIC = 32–64 μg/ml) and **7** (MIC = 8–64 μg/ml) were the most active compounds. Cut-off points for antimicrobial activities were defined as follows: *(i)* For crude extracts: significant activity (MIC < 100 μg/ml), moderate activity (100 < MIC ≤ 625 μg/ml) or weak activity (MIC > 625 μg/ml); *(ii)* For pure compounds: significant activity (MIC <10 μg/mL), moderate activity (10 < MIC ≤ 100 μg/ml), and low activity (MIC > 100 μg/ml) [20, 21]. Hence, the activity recorded herein for the extracts (128 < MIC ≤ 512 μg/ml) and compounds **1** and **7** (10 < MIC ≤ 100 μg/ml) is moderate whereas that of compounds **2, 3, 5a, 6, 8** and **9** (MIC > 100 μg/ml) is low. The lowest MIC value of 8 μg/ml was recorded with compound **7** on *Staphylococcus aureus*, highlighting some medicinal potential for this compound, as the activity on *S. aureus* was equal to that of ampicillin. *S. aureus* is a major cause of community and hospital-associated infection with an estimated mortality of around 7–10% [15, 22]. Moreover, about 2% of patients in Cameroon are infected by *Staphylococcus* spp [14]. Each year, some 500,000 patients in American hospitals contract a staphylococcal infection [15, 22]. Such findings stress the importance of finding an antibiotic against which *Staphylococcus aureus* is sensitive. It can also be noted that the reference antibiotics were in most of the case more active than all studied samples, except on *Vibrio cholerae* NB2, *Vibrio cholerae* PC2 and *Shigella flexneri* where ampicillin was not active. However, these bacterial strains were found to be sensitive to most of the tested samples, suggesting that their administration may represent an alternative treatment against the *V. cholerae*, the causative agent of cholera and *S. flexneri*, the causative agent of shigellosis. Taking into account the medical importance of the tested bacteria, this result can be considered as promising in the perspective of new antibacterial drugs development. Although iridoids (sanshiside methyl ester, sanshiside-D, mussaein, verbascoside, plumieride, protoplumericin A, plumieride acid, plumericin and isoplumericin) have been reported to possess antiviral, antimicrobial, anticancer and antioxidant properties [23–28], this study report for the first time the antibacterial activity of iridoids on MDR clinical multi-drug resistant (MDR) pathogenic bacteria.

**Table 1:** Antibacterial activity (MIC and MBC in μg/ml) of extracts, isolated compounds and reference antibacterial drugs

Extracts/compounds	Inhibition parameters	*Vibrio cholerae* SG24 (1)	*Vibrio cholerae* CO6	*Vibrio cholerae* NB2	*Vibrio cholerae* PC2	*Shigella flexneri* SDINT	*Staphylococcus aureus ATCC 25923*
MeOH extract	MIC	512	256	512	512	256	128
	MBC	512	256	512	512	256	256
	MBC/MIC	1	1	1	1	1	2
*n*-BuOH extract	MIC	512	512	512	512	256	128
	MBC	>512	512	512	>512	512	128
	MBC/MIC	/	1	1	/	2	1
EtOAc extract	MIC	512	128	256	512	256	128
	MBC	512	256	512	512	512	128
	MBC/MIC	1	2	2	1	2	1
Hexane extract	MIC	>512	512	512	512	256	256
	MBC	/	>512	>512	>512	>512	>512
	MBC/MIC	/	/	/	/	/	/
1	MIC	64	64	64	64	32	32
	MBC	128	64	64	64	64	32
	MBC/MIC	2	1	1	1	2	1
2	MIC	256	128	128	256	128	64
	MBC	>256	256	128	256	256	64
	MBC/MIC	/	2	1	1	2	1
3	MIC	>256	128	256	256	256	256
	MBC	/	256	256	>256	>256	>256
	MBC/MIC	/	2	1	/	/	/
5a	MIC	256	128	128	128	128	128
	MBC	>256	256	128	256	128	128
	MBC/MIC	/	2	1	2	1	1
6	MIC	128	256	256	128	32	32
	MBC	256	256	256	256	64	64
	MBC/MIC	2	1	1	2	2	2
7	MIC	32	64	32	32	16	8
	MBC	64	128	32	32	16	8
	MBC/MIC	2	2	1	1	1	1
8	MIC	256	256	128	128	128	64
	MBC	>256	256	>256	256	256	128
	MBC/MIC	/	1	/	2	2	2
9	MIC	128	128	128	256	128	128
	MBC	128	>256	128	256	256	256
	MBC/MIC	1	/	1	1	2	2
Ampicillin	MIC	16	16	>512	>512	>512	8
	MBC	16	16	>512	>512	>512	8
	MBC/MIC	1	1	/	/	/	1
Ciprofloxacin	MIC	8	8	16	16	16	2
	MBC	8	8	16	16	16	2
	MBC/MIC	1	1	1	1	1	1

/ not determined, *MIC* minimum inhibitory concentration, *MBC* minimum bactericidal concentration

The results of Table 1 also showed detectable MBC values for most of the studied samples on the tested bacterial strains. When analysing carefully the MIC and MBC results for the extracts and compounds, it can be noted that MBC/MIC ratios lower than 4 were obtained with these samples on most of the tested microbial species, suggesting that a bactericidal effect could be expected [29, 30].

### Antioxidant activity

**Fig. 2:**
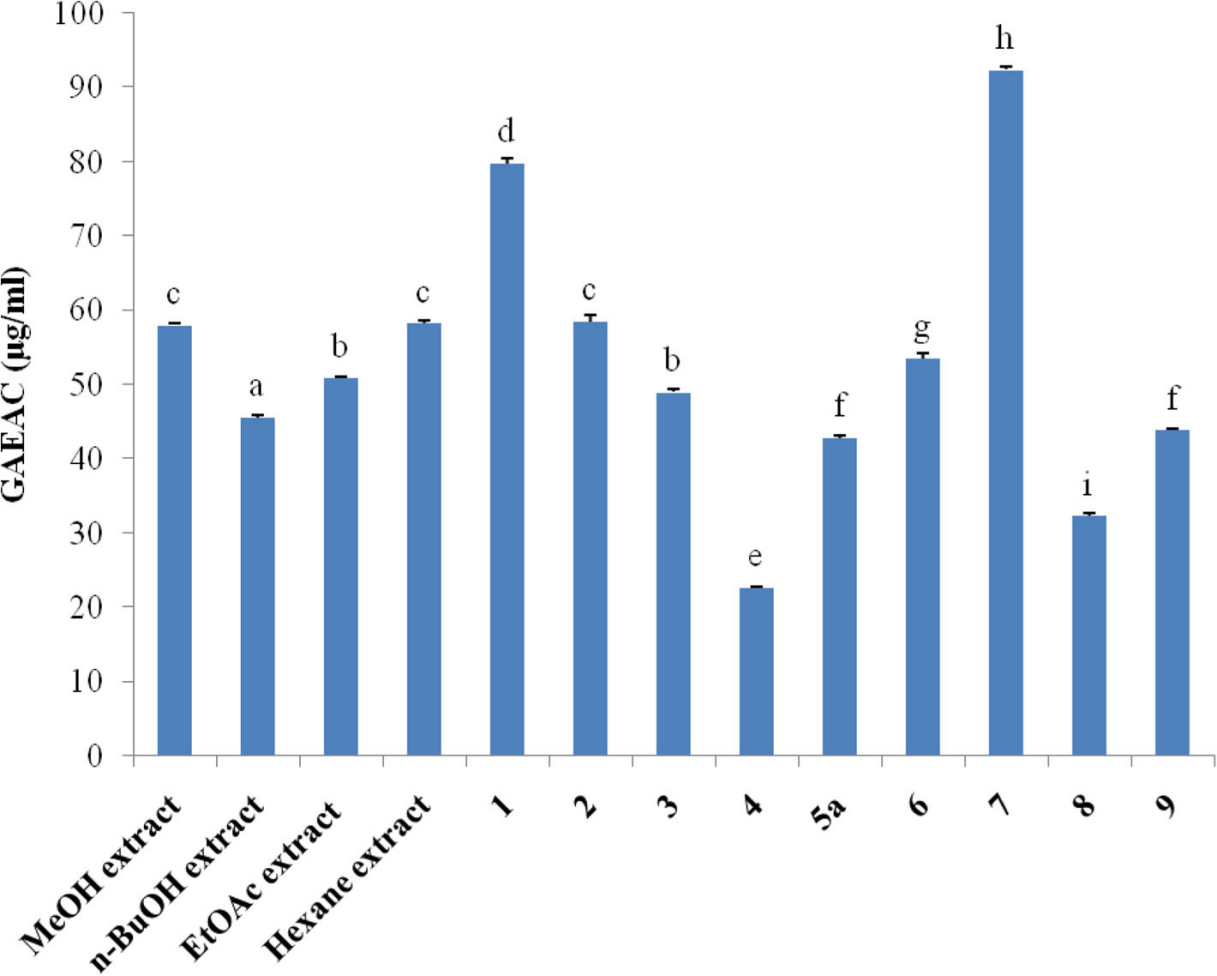
Gallic acid equivalent antioxidant capacity (GEAC; μg/ml) of tested samples. Bars represent the mean ± SD of three independent experiments carried out in triplicate. Letters a-i indicate significant differences between samples according to one way ANOVA and Waller Duncan test; *p* < 0.05. **1:** canthiumoside 1; **2:** canthiumoside 2; **3:** canthiumoside 3; **4:** canthiumoside 4; **5a:** canthiumoside 5a; **6:** shanzhigenin methyl ester; **7:** linearin; **7’:** 1-epilinearin; **8:** mussaenoside; **9:** shanzhiside methyl ester

**Fig. 3:**
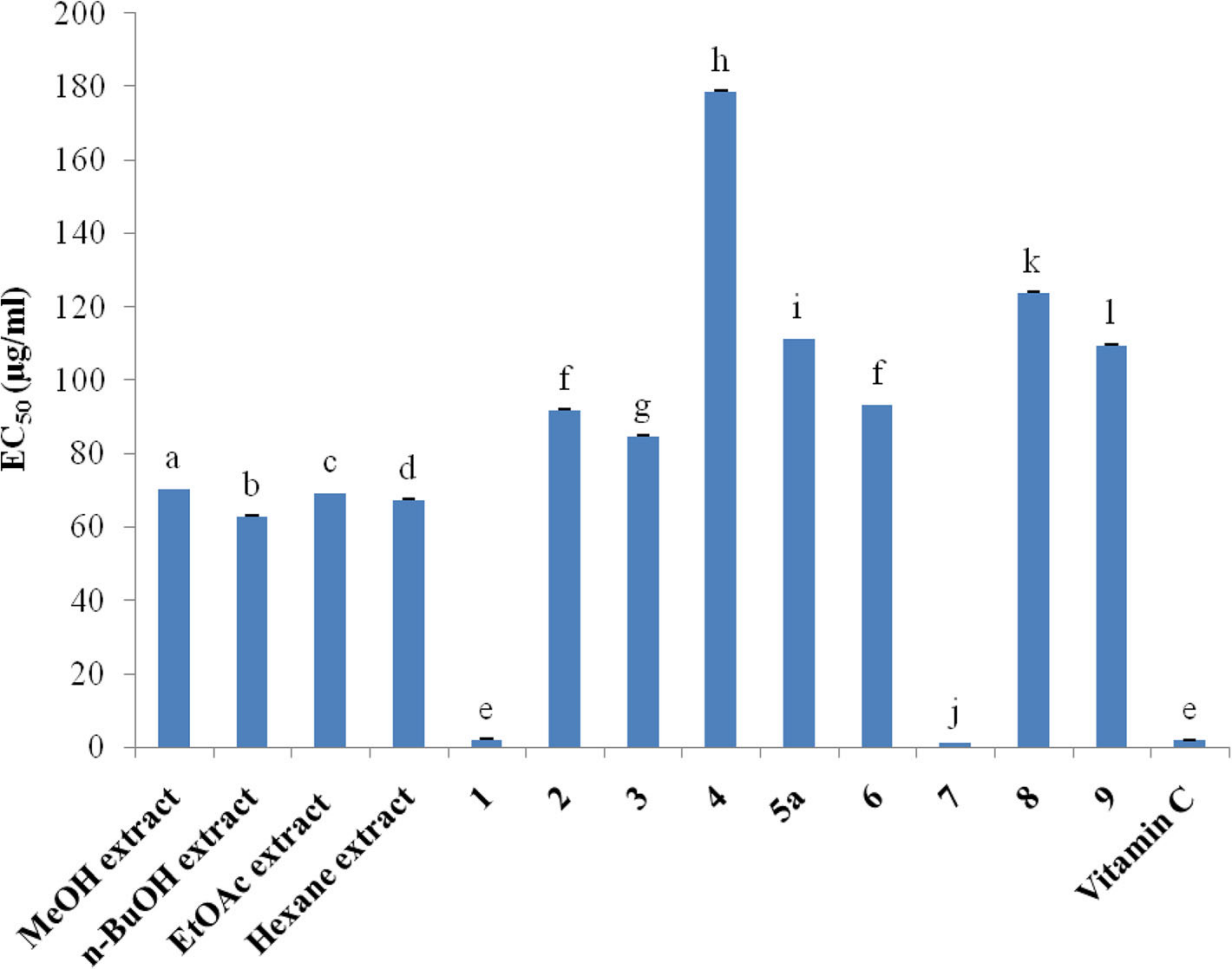
Equivalent concentrations of test samples scavenging 50% of DPPH radical (EC_50_). Bars represent the mean ± SD of three independent experiments carried out in triplicate. Letters a-l indicate significant differences between samples according to one way ANOVA and Waller Duncan test; *p* < 0.05. **1:** canthiumoside 1; **2:** canthiumoside 2; **3:** canthiumoside 3; **4:** canthiumoside 4; **5a:** canthiumoside 5; **6:** shanzhigenin methyl ester; **7:** linearin; **7’:** 1-epilinearin; **8:** mussaenoside; **9:** shanzhiside methyl ester

Plant based antioxidant compounds [31, 32] play a defensive role by preventing the generation of free radicals and hence are extremely beneficial to alleviate the diseases caused by oxidative stress such as cardiovascular diseases, diabetes, inflammation, degenerative diseases, cancer, anemia, and ischemia [33, 34]. In this study, free radical scavenging capacities were measured using DPPH radical and ABTS radical cation. The results are expressed as gallic acid equivalent antioxidant capacity of tested samples (Figs. 2 and 3) and as equivalent concentrations of test samples scavenging 50% of DPPH radical (Fig. 3). Compounds **1** (EC_50_ = 2.03 μg/ml; GAEAC = 79.82 μg/ml) and **7** (EC_50_ = 1.12 μg/ml; GAEAC = 92.35 μg/ml) exerted the greatest activity whereas compound **4** (EC_50_ = 178.57 μg/ml; GAEAC = 22.63 μg/ml) displayed the lowest antioxidant activity in both assays (*p* < 0.05); suggesting that the ability of these samples to scavenge DPPH could also reflect their ability to inhibit the formation of ABTS+. Apart from compounds **1** and **7**, the EC_50_ value of vitamin C is lower than those of the other tested samples, showing that these samples are less active compared with vitamin C. However, the EC_50_ value of compound **7** (EC_50_ = 1.12 μg/ml) is lower than that of standard vitamin C (EC_50_ = 1.74 μg/ml), clearly indicating that this compound is more potent than vitamin C in scavenging free radicals in vitro (Fig. 3). Moreover, the EC_50_ value of compound **1** (EC_50_ = 2.03 μg/ml) is comparable to that of vitamin C (EC_50_ = 1.74 μg/ml). In all, the DPPH and ABTS scavenging activities in this study indicated that compounds **1** and **7** belonging to iridoids were potent antioxidants. Previous studies reported the antioxidant activities of some iridoids from plant origin [35–37]. Hence, the antioxidant activity of extracts in this study may be due to the presence of iridoids and phenolic compounds that are capable of donating hydrogen to a free radical in order to remove odd electron, which is responsible for the radical’s reactivity [28, 38].

### Hemolytic activity

In this study, none of the tested samples showed hemolytic activities against human red blood cells at concentrations up to 512 μg/ml for the extracts and 256 μg/ml for pure compounds (results not shown) indicating that it is non-toxic to normal cells.

## Conclusion

Our results demonstrated that compounds **1** and **7** under investigation were potent antibacterials and DPPH/ABTS·^+^ radical scavengers.

## Abbreviations

^13^C-NMR: Thirteen carbon nuclear magnetic resonance
^1^H NMR: Proton nuclear magnetic resonance
ABTS: 2,2’-azinobis(3-ethylbenzothiazoline-6-sulfonic acid)
ATCC: American type culture collection
COSY: Correlation spectroscopy
DMSO: Dimethylsulfoxide
DPPH: 1,1-diphenyl-2-picrylhydrazyl radical
EC_50_: Concentration scavenging 50% DPPH radicals
EtOAc: Ethyl acetate
GAEAC: Gallic acid equivalent antioxidant capacity
HMBC: Heteronuclear multiple bond connectivities
HR-TOFESIMS: High-resolution time of flight electrospray ionization mass spectrometry
HSQC: The heteronuclear single quantum coherence
IP: Institut pasteur
IR: Infra-red
MBC: Minimum bactericidal concentration
MeOH: Methanol
MHA: Mueller Hinton agar
MHB: Mueller Hinton broth
MIC: Minimum inhibitory concentration
NA: Nutrient agar
n-BuOH: n-Butanol
NMR: Nuclear magnetic resonance
NOESY: Nuclear overhauser effect spectroscopy
SRF/CAM: Section de réserve forestière du Cameroun
